# P-2058. Neutralizing Antibody Kinetics and Projected Immunity up to 12-months after COVID Vaccination in Nursing Home Residents

**DOI:** 10.1093/ofid/ofae631.2214

**Published:** 2025-01-29

**Authors:** Jürgen Bosch, Alexandra Paxitzis, Oladayo A Oyebanji, Olajide Olagunju, Michael C Payne, Vaishnavi Ragavapuram, Nicholas Sundheimer, Yi Cao, Yasin Abul, Clare Nugent, Alejandro Balazs, Christopher King, Stefan Gravenstein, Brigid WIlson, David Canaday

**Affiliations:** Case Western Reserve University, Cleveland, Ohio; CWRU, Cleveland, Ohio; Case Western Reserve University, Cleveland, Ohio; Case Western Reserve University, Cleveland, Ohio; Case Western Reserve University School of Medicine, University Heights, Ohio; CWRU, Cleveland, Ohio; Case Western Reserve University, Cleveland, Ohio; Ragon, Cambridge, Massachusetts; Brown University, Providence, Rhode Island; Brown, Providance, Rhode Island; Ragon, Cambridge, Massachusetts; Case Western Reserve University, Cleveland, Ohio; Brown University, Providence, Rhode Island; Cleveland VA, Cleveland, Ohio; VA Northeast Ohio Healthcare System, Cleveland, Ohio

## Abstract

**Background:**

Immune response and memory changes with aging and disease, features that may be amplified in long-term care populations. We evaluated the kinetics of immunity following vaccination and infection in nursing home residents (NHR).

**Figure:**
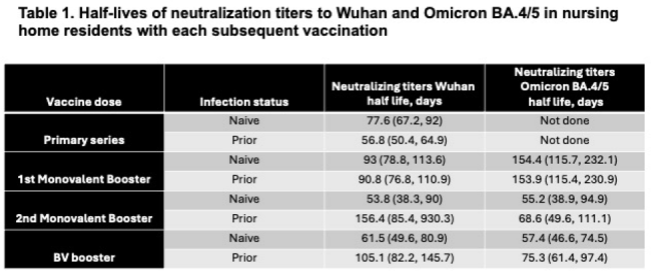

**Methods:**

In a cohort of NHR with continuous enrollment since 2020, we drew blood before and after mRNA COVID-19 vaccines and SARS-COV-2 infection. We identified prior and breakthrough infections and the intervals between vaccinations and samples to describe decay kinetics of neutralizing titers over time. Assuming a peak response occurring 14 days after each dose, we estimated mixed-effects exponential decay models, stratified by dose and prior infection and assuming a random intercept for each subject. From these models, we estimated peak value and rates of decay for Wuhan and Omicron BA.4/5 neutralizing titers.

**Figure:**
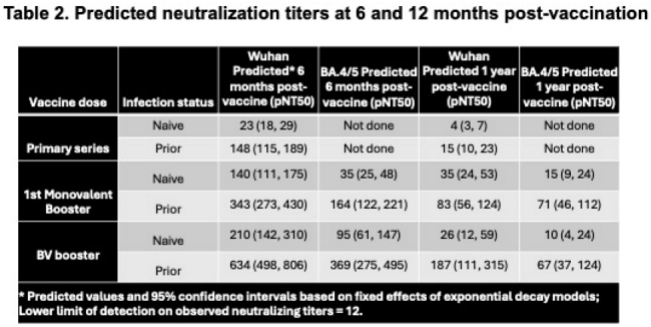

**Results:**

Our analysis cohort followed 412 NHR (median (IQR) age = 76 (69, 86); 49% female). Among infection-naive NHR, the estimated Wuhan rate of decay did not differ significantly across doses. Prior infected NHR showed significantly longer half-lives after all booster doses than with the primary series (Figure 1, Table 1). Decay models of Omicron BA.4/5 neutralizing titers following booster doses showed significant increases in peak titer for each subsequent booster among both the naive and prior infection subgroups. The decay rates increased significantly over the first monovalent booster, but no differences in rates were detected between infection-naive and prior infection subgroups (Figure 2). The estimated half-lives across strain, dose, and infection subgroups ranged from roughly 50 to 150 days; the differences in peak and decay are reflected in the predicted titers at 6 and 12 months after vaccination (Table 2).

**Figure:**
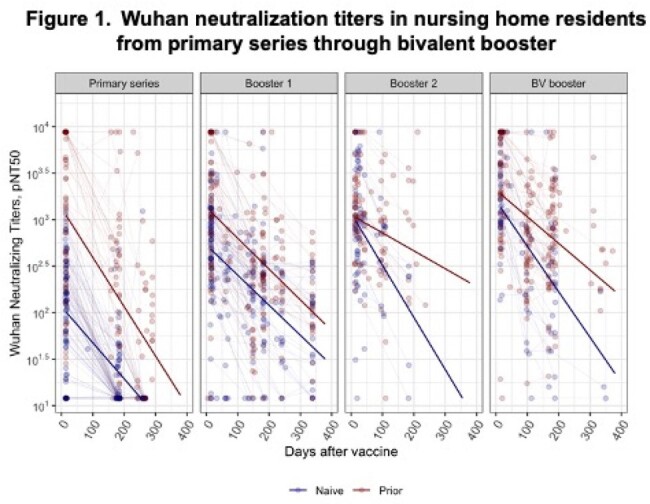

**Conclusion:**

Following primary series and booster doses, NHR with prior infection enjoy both higher peaks and a similar or slower decline in neutralizing titers than naive NHR, demonstrating the benefits of hybrid immunity with subsequent vaccinations. Neutralizing titers decline sufficiently fast to warrant updating vaccines before the prior dose anniversary. Understanding the kinetics of serial doses within this population can assist in vaccine planning and priorities among NHR.

**Figure:**
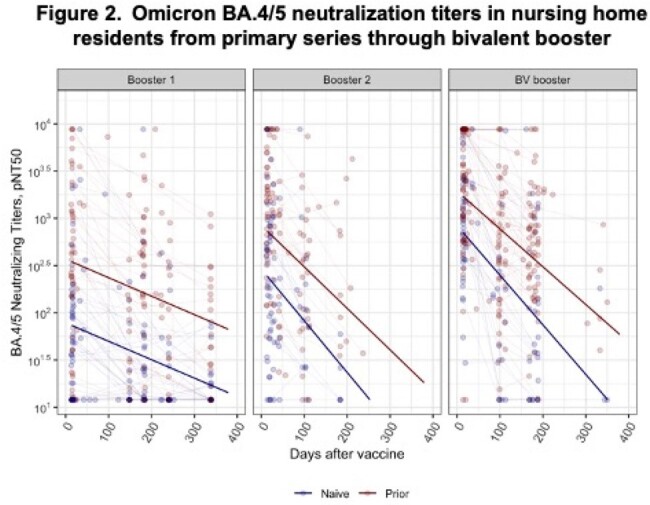

**Disclosures:**

Yasin Abul, MD, Moderna: Grant/Research Support|Moderna, Abt, CDC: Grant/Research Support Alejandro Balazs, PhD, Cure Systems LLC: Ownership Interest Stefan Gravenstein, MD, MPH, CDC: Advisor/Consultant|CDC: Grant/Research Support|Genentech: Advisor/Consultant|Genentech: Grant/Research Support|Genentech: Honoraria|GlaxoSmithKline: Advisor/Consultant|GlaxoSmithKline: Grant/Research Support|GlaxoSmithKline: Honoraria|Janssen: Advisor/Consultant|Janssen: Grant/Research Support|Janssen: Honoraria|Moderna: Advisor/Consultant|Moderna: Grant/Research Support|Moderna: Honoraria|NIH: Grant/Research Support|Pfizer: Advisor/Consultant|Pfizer: Grant/Research Support|Pfizer: Honoraria|Sanofi: Advisor/Consultant|Sanofi: Grant/Research Support|Sanofi: Honoraria|Seqirus: Advisor/Consultant David Canaday, MD, Moderna: Grant/Research Support|Pfizer: Grant/Research Support

